# Supplementary material for: Functional dissection of H3K4 methyltransferases reveals distinct catalytic and non-catalytic roles in C. elegans development
Source: Development. 2025 Nov 3;152(21):dev204924. doi: 10.1242/dev.204924 (PMC12633793; doi:10.1242/dev.204924)
Supplement: Supplementary information [file develop-152-204924-s1.pdf]

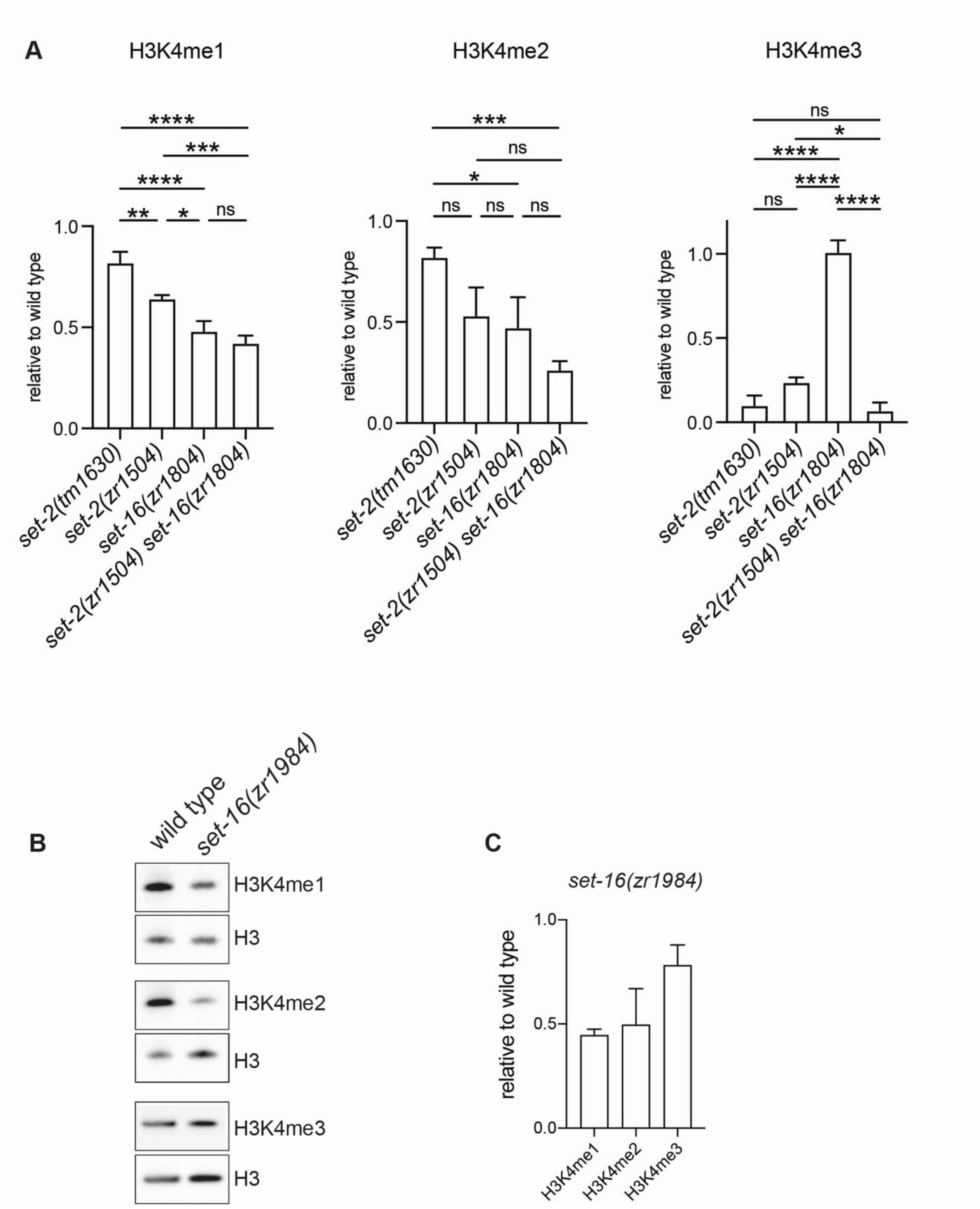


**Fig. S1. SET mutants catalytic activities.** (A) Quantification of H3K4me1/2/3 levels in mutants. Levels reported are relative to wild type and are presented as mean +/- s.e.m. of at least three independent biological experiments. Statistical analysis was performed using one-way ANOVA followed by Tukey's multiple comparison *p<0.05, **p<0.01, ***p<0.001, ****p<0.0001 and ns (not significant) (B) Representative western blots of lysates from mixed embryos of *set-16(zr1984)* carrying a mutation in the SET (R2389W), probed with antibodies specific for H3K4me1/2/3. H3 is used as loading control. (C) Quantification of H3K4me1/2/3 levels in *set-16(zr1984)*. Levels reported are relative to wild type and are presented as mean +/- s.e.m. of at least three independent biological experiments.

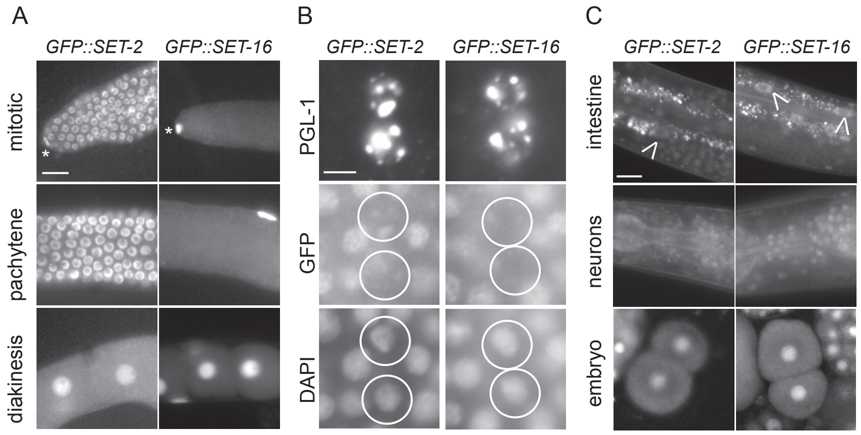


**Fig. S2. SET-2 and SET-16 expression patterns.** Representative images of GFP-tagged SET-2 and SET-16 expression in (A) indicated compartments of the germline, (B) primordial germ cells precursor (in white circles, identified by PGL-1 and DAPI staining) and (C) Indicated somatic tissues and two-cell embryos. in B, anti-GFP antibody was used. Asterisks depict distal tip cells, arrowheads intestinal cells. In A and C, scale bar is 10µm, in B, 5µm.

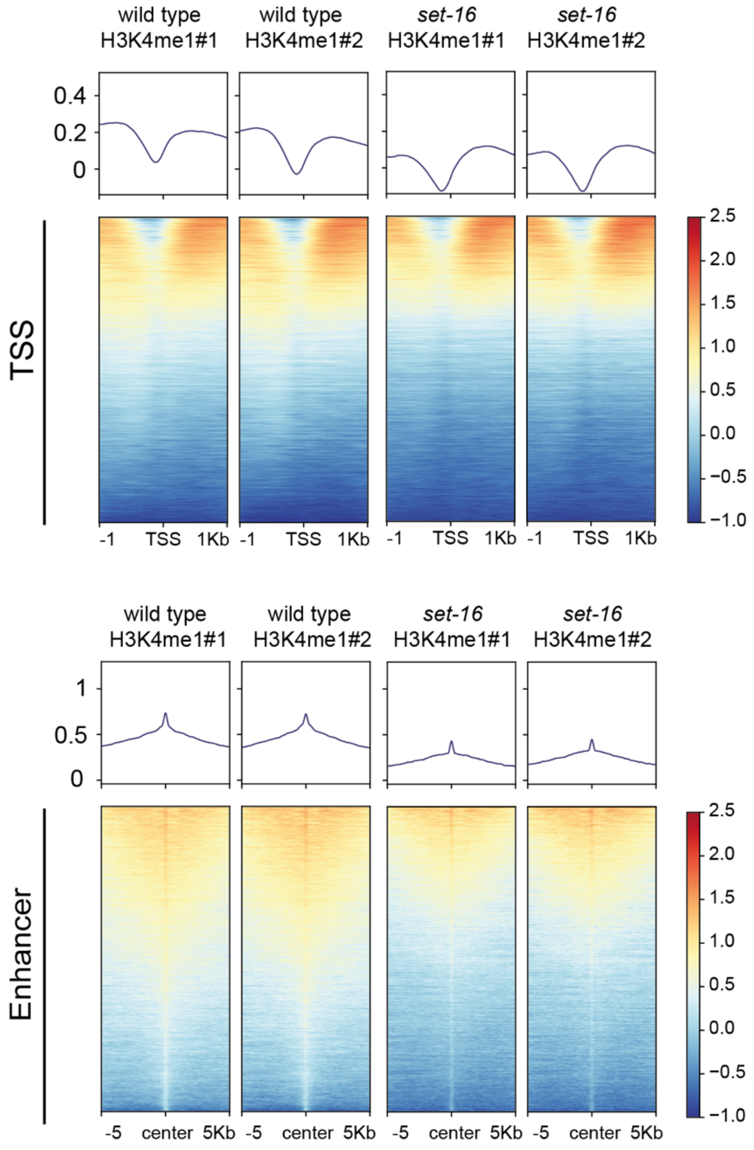


**Fig. S3. Genome-wide analysis of SET-16 catalytic activities on H3K4me1 level.** (A) Heatmaps of H3K4me1 ChIP-seq signal at Transcription Start Sites (TSS +/-1Kb) (top) and putative enhancers (+/-5Kb from centre) (bottom) in wild type and *set-16(zr1804).* Two biological independent experiments are shown.

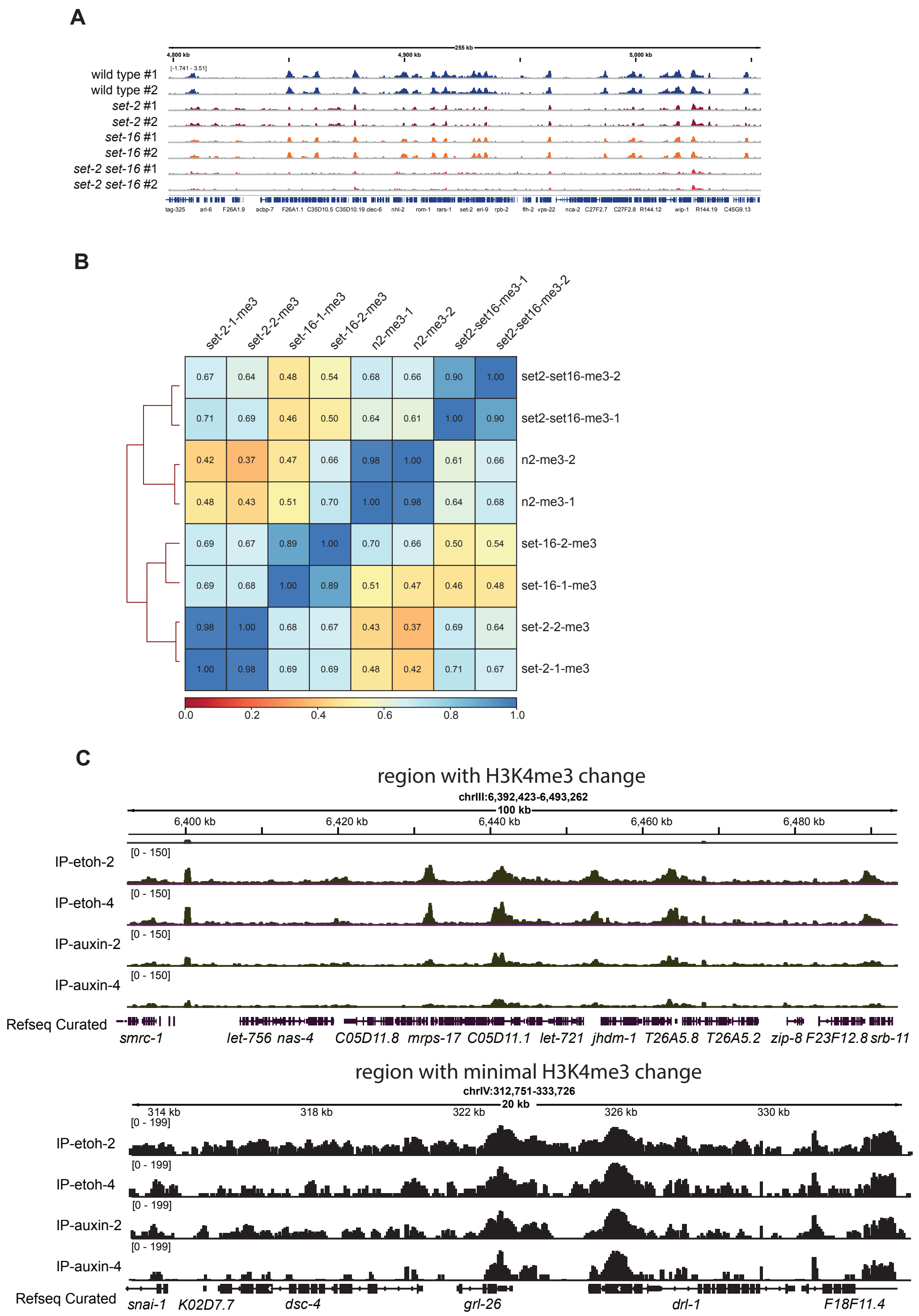


**Fig. S4. Genome-wide occupancy of H3K4me3.** (A) Representative track view of ChIP-seq profiles in indicated genetic backgrounds. (B) Spearman correlation of reads counts of H3K4me3 ChIP performed in indicated genetic backgrounds. (C) Representative track view of ChIP-seq profiles with and without auxin exposure – Two regions are shown, representing different degrees of auxin effect on H3K4me3 levels. Two replicas are shown

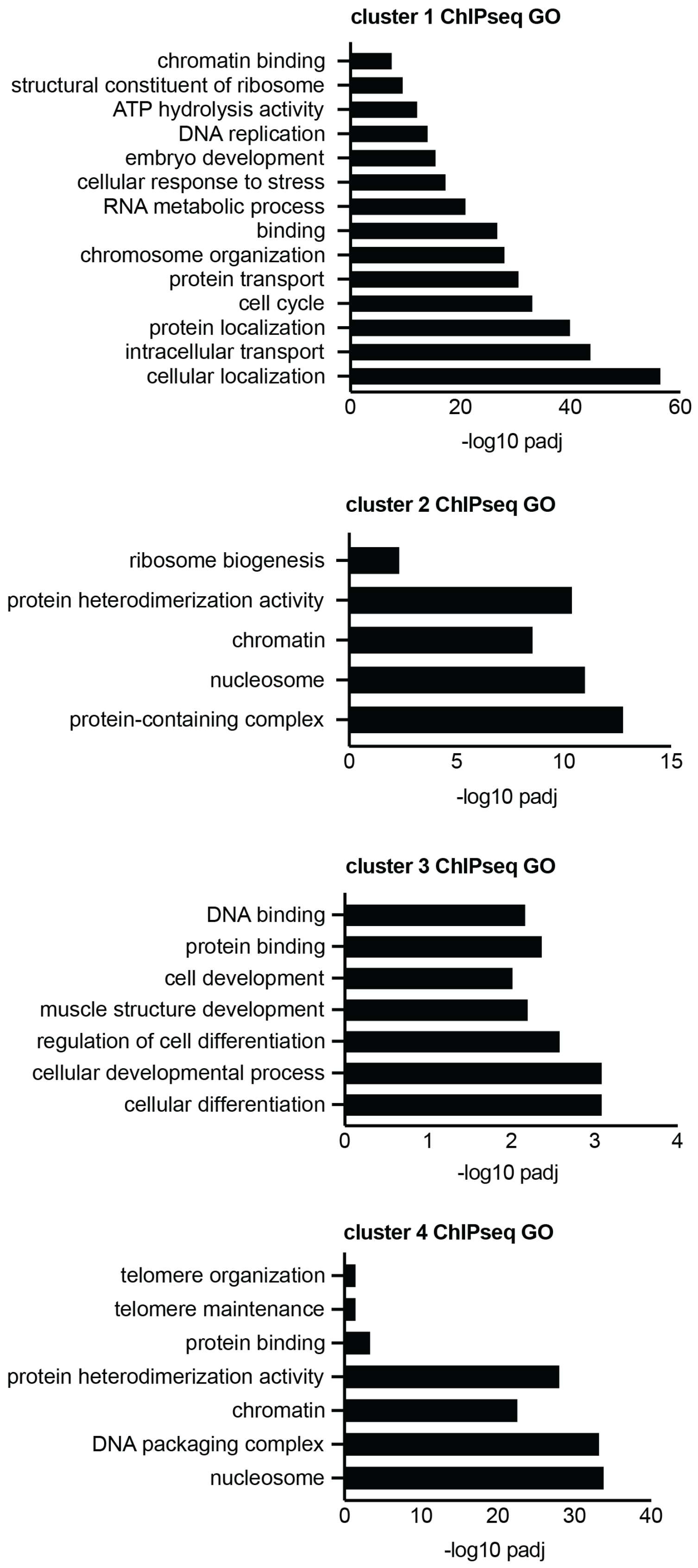


**Fig. S5. Gene ontology analysis of H3K4me3 genes clusters.** Top categories of biological processes (BP) identified as enriched in four different gene clusters with distinct H3K4me3 patterns among *set-2(zr1504), set-16(zr1804)* and *set-2(zr1504) set-16(zr1804)* double mutants, illustrated in Fig. 4D and Fig.4E. GO analysis was performed with g-profiler (P-value<0.05 with Bonferroni correction) and GO terms are presented as -log10padj.

Development • Supplementary information

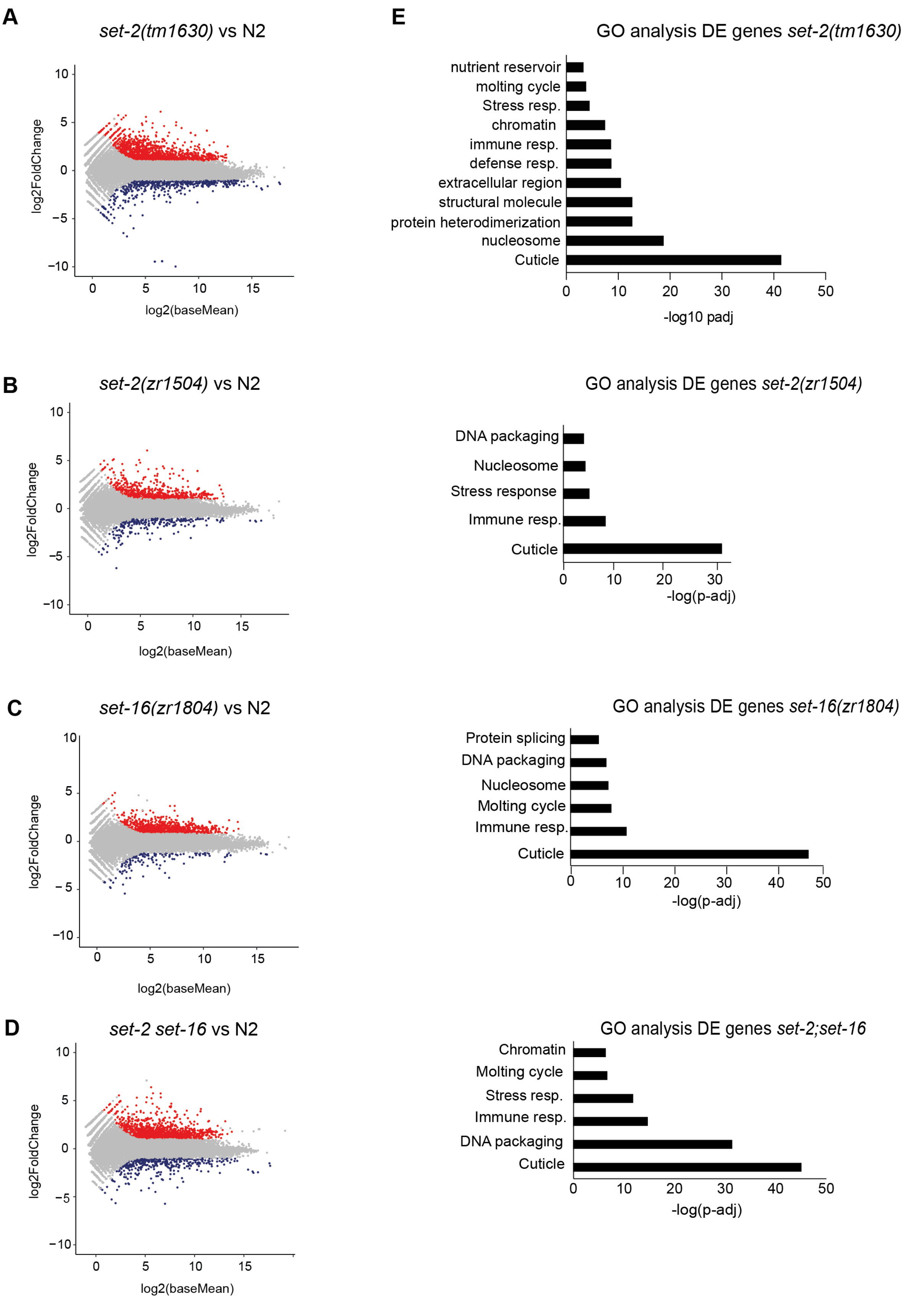


**Fig. S6. Gene expression in *set-2, set-16 and set2 set-16* mutants.** (A-D) MA plots illustrating DE genes in indicated genetic backgrounds, compared to wild-type (N2). (E) Gene ontology analysis of differentially expressed genes in *set-2(zr1504), set-16(zr1804)* and *set-2(zr1504) set-16(zr1804)* double mutants. GO analysis was performed with g-profiler (P-value<0.05 with Bonferroni correction) and GO terms are presented as -log10padj.

Development • Supplementary information

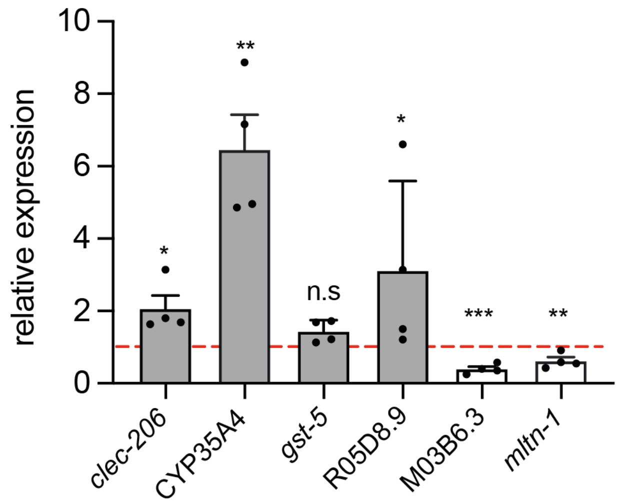


**Fig. S7. Validation of SET-2 immediate transcriptional targets by quantitative PCR**
mRNA levels of some of DE genes identified by RNA-seq in auxin-treated animals analysed by qPCR. Expression is normalized to *pmp-3* and *cdc-42* levels. Data are average of four independent biological replicas represented as mean +/- s.e.m. Statistical significance was evaluated using unpaired student's t-test. *p<0.05, **p<0.01 and ***p<0.001.

**Table S1. ChIP in all mutant**

Available for download at
https://journals.biologists.com/dev/article-lookup/doi/10.1242/dev.204924#supplementary-data

**Table S2. RNAseq all mutants**

Available for download at
https://journals.biologists.com/dev/article-lookup/doi/10.1242/dev.204924#supplementary-data

**Table S3. ChIP and RNAseq auxin treatment**

Available for download at
https://journals.biologists.com/dev/article-lookup/doi/10.1242/dev.204924#supplementary-data

## Table S4. Strain list

| Genotype/Description | Strains |
|---|---|
| | |
| N2 | wild-type, Bristol |
| *oyls14[sra-6p::GFP]* | ZR1153 |
| *set-2(tm1630)* | ZR1032 |
| *set-2(tm1630);oyls14* | ZR976 |
| *set-2(zr1504)* | ZR1140 |
| *set-2(zr1504);oyls14* | ZR1141 |
| *set-16(zr1804)* | ZR1142 |
| *set-16(zr1804);oyls14* | ZR1143 |
| *set-16(zr1984)* | ZR1126 |
| *set-16(n4526)/qC1[dpy-19(e1259) glp-1(q339)]* | MT14615 |
| *set-2(zr1504) set-16(zr1804)* | ZR1267 |
| *set-2(zr1504) set-16(zr1804);oyls14* | ZR1157 |
| *set-2(syb1554) gfp::set-2* | PHX1554 |
| *set-16(syb1762) gfp::set-16* | PHX1762 |
| *set-2(syb4167) Degron::GFP::set-2* | PHX4167 |
| *ieSi38 [sun-1p::TIR1::mRuby::sun-1 3'UTR + Cbr-unc-119(+)]* | CA1199 |
| *ieSi57 [eft-3p::TIR1::mRuby::unc-54 3'UTR + Cbr-unc-119(+)]* | CA1200 |
| *set-2(syb4167); Oyls14; IeSi38; IeSi57* | ZR1178 |

Development • Supplementary information
